# No impact of hygienic behavior and viral coinfection on the development of European foulbrood in honey bee (*Apis mellifera*) colonies during blueberry pollination in Michigan

**DOI:** 10.1093/jisesa/iead094

**Published:** 2023-12-06

**Authors:** Peter D Fowler, Declan C Schroeder, Jessica L Kevill, Meghan O G Milbrath

**Affiliations:** Comparative Medicine and Integrative Biology, Michigan State University, East Lansing, MI 48824,USA; Department of Veterinary Population Medicine, University of Minnesota, St. Paul, MN, USA; School of Biological Sciences, University of Reading, Reading, UK; Department of Veterinary Population Medicine, University of Minnesota, St. Paul, MN, USA; Centre for Environmental Biotechnology, School of Natural Sciences, Bangor University, Bangor LL57 2UW, Gwynedd, UK; Department of Entomology, Michigan State University, Pollinator Performance Center, 4090 N. College Road, RM 100, Lansing, MI 48910, USA

**Keywords:** European foulbrood, honey bee, hygienic behavior, deformed wing virus

## Abstract

European foulbrood (EFB) is a severe disease of honey bee (*Apis mellifera*) larvae caused by the bacterium Linnaeus [Hymenoptera: Apidae]) *Melissococcus plutonius* (ex White) Bailey and Collins (Lactobacillales: Enterococcaceae). Many beekeepers in North America report severe EFB following blueberry pollination, but it is not clear what factors during pollination are related to clinical disease. Additionally, the impact that other factors such as viral load and hygienic behavior have on EFB has not been studied. In Spring of 2020 we enrolled 60 commercial honey bee colonies in a prospective cohort study. Colonies were inspected 3 times over the season with hive metrics and samples taken for viral testing. Each colony was tested for hygienic behavior twice and the score was averaged. Viral loads were determined by qPCR for deformed wing virus (DWV) A and B. We found no statistical difference in the EFB prevalence or severity between the 2 yards at any timepoint; 50% (*n* = 16) of the colonies in the holding yard and 63% (*n* = 17) in blueberry developed moderate to severe EFB over the study period. When colonies from both yards were pooled, we found no relationship between viral load or hygienic behavior and development of EFB. These results suggest that other factors may be responsible for driving EFB virulence and hygienic behavior is not likely helpful in managing this disease.

## Introduction

The western honey bee (*Apis mellifera* Linnaeus [Hymenoptera: Apidae]) plays a critical role in United States agriculture providing an estimated 18 billion USD in value annually (Keel 2022). Parasites and diseases have been a burden on the health of the honey bee industry in recent years. According to recent data collected by Bee Informed Partnership, US commercial beekeepers lost an estimated 46.1% of their colonies from 2019 to 2020 (Bruckner et al. 2022). With a significant portion of our crops reliant on these animals for production, improving the health of the beekeeping industry remains a top priority for long-term sustainability of our agriculture system.

One of the most prevalent and serious diseases impacting colony health in North America is European foulbrood, commonly referred to as EFB (de León-Door et al. 2018, Laate et al. 2020, Grant et al. 2021, Milbrath et al. 2021). The economic impact of EFB disease has been estimated to be between $300 and $500 USD per hive in reduced production and increased management costs (Laate et al. 2020), an unsustainable burden to an already struggling beekeeping industry. The only approved treatment for this disease in the United States is the antibiotic Oxytetracycline, and the labor-intensive treatment protocol and long honey withdrawal times make its use impractical for many beekeepers (Richards et al. 2021). Additionally, a recent study in Canada found some strains of the bacterium that causes EFB may be developing resistance (Masood et al. 2022), making disease prevention strategies critical for the sustained health of the industry.

European foulbrood is caused by the bacterium *Melissococcus plutonius* (ex White), Bailey and Collins (Lactobaccillus: Enterococcaceae) (McKee et al. 2004), and despite being discovered more than a century ago (White 1912), little is known about its pathogenesis (Grossar et al. 2020). Honey bee larvae infected with this pathogen can experience high rates of mortality, but, *M. plutonius* is also commonly found in asymptomatic colonies (Belloy et al. 2007, Roetschi et al. 2008, Budge et al. 2010, Erban et al. 2016). Disease onset is known to be seasonal in nature with many colonies recovering after honey flow (Phil1ips 1918, White 1920). This difference in colony outcomes has led many to consider the role that other factors play in driving virulence, such as host microbiome composition (Floyd et al. 2020), pesticide exposure (Wood et al. 2020), nutrition (Wardell 1982) or climate conditions (Grant et al, 2021) but so far, no specific factor has been confirmed to be implicated.

Deformed wing virus (DWV) is a widespread virus known for affecting pupal development and is known to be vectored by *Varroa destructor* (Mesostigmata: Varroidae) (Lanzi et al. 2006). Two dominant genotypes of this virus, DWV-A and DWV-B, are commonly found in mite infested colonies but their relationship to colony health is the subject of ongoing study (Highfield et al. 2009, de Miranda and Genersch 2010, Schroeder and Martin 2012, Penn et al. 2022, Piou et al. 2022). Subclinical DWV infections have been shown to result in impaired cognitive function, foraging performance, and survival in adult bees (Iqbal and Mueller 2007, Benaets et al. 2017, Gisder et al. 2018, Traniello et al. 2020). Stress associated with this disease may leave colonies susceptible to other infections. Because EFB has long been associated with factors that increase stress and may weaken the colony, we hypothesized that colonies with high viral loads may be more susceptible to developing disease in the presence of *M. plutonius.* Additionally, the reduced foraging performance and impaired cognitive function associated with DWV may impact nurse bee care of developing larvae adding to the nutritional deficit thought to trigger EFB symptoms. Alternatively, we hypothesized that colonies weakened by EFB may be more susceptible to viral replication resulting in higher viral loads in severely affected colonies.

Hygienic behavior is a widely studied genetic trait of honey bees associated with uncapping and removal of dead brood from the colony (Spivak and Gilliam 1998, Lapidge et al. 2002). This behavior in honey bee colonies has been found to help with the management of another bacterial brood disease, American foulbrood (Spivak and Gilliam 1998, Spivak and Reuter 2001). Analysis of changes in chemical bouquet in *M. plutonius* infected larvae when compared with healthy larvae suggests that olfactory identification of infected larvae is possible but whether this initiates hygienic behavior or the mitigation of EFB remains unclear (Kathe et al. 2021).

In this study we seek to determine what role hygienic behavior and viral coinfection may play on the development of EFB. We hypothesized that honey bee colonies under increased stress due to viral coinfections would be more susceptible to developing EFB. Additionally, we hypothesized that colonies with high levels of hygienic behavior would be less susceptible to EFB.

## Methods

In May of 2020 we enrolled 60 hives from a large commercial beekeeping operation in Southern Michigan in a prospective cohort study. The hives were split into 2 groups of 30, representing 2 common scenarios for Spring bees. The first group was composed of established colonies that had been growing in Florida and were brought to blueberry fields in Southeastern Michigan to fulfill pollination contracts. The second group consisted of small colonies that had been recently made from splits and were kept in a large holding yard away from crop pollination but in the same region of Michigan. All colonies were from a single commercial operation and had previously shared in the same migration route from Florida to California for almond pollination and back to Florida for splitting. Both yards contained other colonies that were not part of this study. In both yards, we selected only colonies that were queenright as indicated by the presence of eggs and the absence of queen cells. The colonies were managed according to standard methods by the collaborating beekeeper regarding feeding and mite treatment. All hives consisted of 2 deep boxes at the beginning of the trial, and a medium box was added to some colonies midway through the trial, as needed. Of the 60 colonies enrolled, 53 were included in this analysis, 27 colonies in the blueberry yard and 26 colonies in the holding yard. One colony died during the study for unknown reasons and 6 were excluded because of a lack of brood.

### Inspections

Colonies were inspected 3 times: once at enrollment, which occurred at the beginning of blueberry bloom (4–6 May 2020); once at the end of blueberry bloom (5–6 June); and once approximately 2 wk post-bloom, when the blueberry colonies had been moved to watermelon pollination (23–24 June). At each health inspection we recorded the following data: queen status, cluster size, frames of brood, visual signs of EFB, and visual signs of other diseases. Colonies were considered queenright if queen-laid eggs were seen. Cluster size was recorded by estimating the frames covered by bees to the nearest half frame, observing the top of each box during normal daytime activity, between 11 AM and 3 PM, and in similar weather conditions to ensure counts could be compared from hive to hive. Each frame of brood that was at least half covered with eggs, larvae, or capped brood was counted to estimate the size of the brood nest. If only 1 side of a frame contained brood, it was considered a half frame of brood. A medium box was added to most hives before the second inspection, and the counts for both cluster size and frames of brood were adjusted to accommodate frame size, with medium frames multiplied by a factor of 0.67 to reflect that they consist of approximately 67% of the cells present in a deep frame. Approximately 150 adult nurse bees were collected from each colony at each timepoint using a sterile 60-ml centrifuge tube and stored at 4 °C in the field and transferred to −20 °C until processing. Thirty of these were used immediately for viral testing as described below and the remainder were held at −20 °C for confirmatory PCR. To determine the impact of EFB on colony growth, the percent change in cluster size was calculated for each colony between the first and last timepoint. Four colonies that were queen-less at the final inspection were removed from this analysis to remove the confounding impact this might have on colony growth.

To ensure that damage from the parasite *V. destructor* was not a confounding factor, colonies were managed for mites by the cooperating beekeeper using the same mite treatment across all colonies. Varroa mite loads were measured at the final inspection by a professional Bee Informed Partnership Tech Transfer Team member using an alcohol wash on 300 nurse bees. Percent infestation was reported as the number of mites seen in the wash, divided by 3 (mites per 100 bees). Mite levels were considered low (less than 3 mites per 100 adult nurse bees) in all hives at the final timepoint and were not considered to affect final outcomes (mite levels reported in Supplementary Material).

### Hygienic Testing

Hygienic testing was performed 2 times on each hive using a standard freeze-kill assay (Spivak and Reuter, 1998), once at enrollment (4–5th May) and again a month later (5th–6th June). Briefly, we selected a frame with at least a 3” diameter patch of capped brood that was mainly in the white eye and pink eye stages. A 3” PVC tube with a beveled edge, which encompasses 160 cells, was pressed into a single patch of closed brood, using water to create a tight seal. Liquid nitrogen (approximately 300 ml) was poured into the tube and allowed to evaporate, freezing and killing the capped brood underneath. After the liquid nitrogen had dissipated the tube was removed and the number of currently open cells within the circle of frozen brood was counted and the frame returned to the hive. This number was subtracted from 160 to get the starting number of dead capped brood. After 24 h, the frame was retrieved, and we recorded the number of cells in which the dead pupa was either partially or completely removed. The hygienic value and partial hygienic value was calculated by determining the percent of dead capped brood fully removed (full hygienic score) or fully and partially removed (partial hygienic score). We performed the same test a second time 1 month later (June 2020). Both the full hygienic value and the partial hygienic value for each colony was calculated by averaging the scores from both tests. For analysis, only the average partial hygienic score was used as this value represents a more detailed analysis of uncapping in addition to removal. To measure the impact of hygienic behavior on the development of EFB we only included colonies in the analysis that were exposed to the pathogen; *M. plutonius* was found viable in adult nurse bees (as described below). Full hygienic scores and figures are included in the Supplementary Material but did not yield any significant differences.

### European Foulbrood Characterization

European foulbrood disease severity was estimated by visual inspection. At each health inspection we closely examined both sides of each brood frame. Third to fifth instar larvae and capped brood were closely examined to visualize the following signs of EFB: twisted/malpositioned larvae, discoloration, visible trachea, melted appearance, rubbery scale, or sunken cappings. EFB disease severity was classified according to a previously published classification system (Roetschi et al. 2008, Grant et al. 2021) with a modified cutoff of 100 diseased cells for severe disease: No disease: no signs of diseased brood, possible disease: 1–10 cells showing symptoms, moderate disease: 11–100 cells showing symptoms, severe disease: >100 cells showing symptoms. To simplify analysis, colonies with 10 cells or fewer showing symptoms were considered healthy, while colonies with more than 10 cells affected were considered diseased. All colonies diagnosed with EFB were confirmed by culture of *M. plutonius* from adult nurse bees, followed by duplex PCR as described by Arai et al. 2012 (see below). Colonies with more than 10 cells showing symptoms were considered diseased with EFB. There were only 2 colonies that recovered (went from > 10 cells with signs of disease to <10 cells), 1 was in blueberry pollination and the other was in the holding yard, and both started healthy at timepoint 1 and became diseased at timepoint 2 but were healthy at timepoint 3. Because there was no difference in EFB incidence between the 2 yards at any timepoint, and because the average hygienic score and viral loads did not differ statistically between the 2 yards (Table 1), for analysis, colonies from both yards were combined (*n* = 53).

**Table 1.** There was no statistically significant difference in the incidence of EFB, or the levels of DWV-A or DWV-B between the blueberry yard and the holding yard at any timepoint. EFB is reported in percentage of colonies with clinical disease (more than 10 cells showing symptoms). DWV is reported in the average viral load in Log10 viral copies per adult bee for each yard. Overall is the percentage of colonies in each yard that showed signs of EFB at any timepoint. For DWV, overall is the average of all measurements for each yard

		Timepoint 1 (4th–6th May)	Timepoint 2 (5th–6th June)	Timepoint 3 (23rd–24th June)	Overall
EFB—Percent of colonies with disease	Blueberry	7% (2/27)	37% (10/27)	63% (17/27)	67% (18/27)
	Holding	4% (1/26)	35% (9/26)	50% (13/26)	58% (15/26)
DWV-A—Average viral load per yard	Blueberry	8.7 (*n* = 26)	9.3 (*n* = 25)	8.8 (*n* = 27)	8.9 (*n* = 78)
	Holding	9.1 (*n* = 25)	8.7 (*n* = 26)	8.1 (*n* = 25)	8.6 (*n* = 76)
DWV-B—Average viral load per yard	Blueberry	8.9 (*n* = 25)	9.1 (*n* = 24)	9.0 (*n* = 25)	8.9 (*n* = 74)
	Holding	8.3 (*n* = 21)	8.9 (*n* = 25)	8.4 (*n* = 25)	8.6 (*n* = 70)

### Quantification of DWV-A and DWV-B Viral Load

From each colony, at each of the 3 timepoints; T1 (4th–6th May), T2 (5th–6th June), T3 (23rd–24th June), 30 adult nurse bees were frozen in liquid nitrogen and ground into a fine powder and 30 mg of this homogenate underwent total RNA extraction using the RNeasy Mini Kit (QIAGEN) according to the manufacturer’s instructions. RNA was quantified using a NanoDrop spectrophotometer and were diluted to 50 ng/µl. We used the ABC assay (Kevill et al. 2017), but omitted DWV-C detection because DWV-C was not present in our study region (Kevill et al. 2019). The ABC assay reports on the conserved *RdRp* gene and represents the 3’ region of the DWV master genomes only. Real-time PCR was performed using a Sensifast SYBR No-Rox One Step Kit (Bioline) with primers for DWV-A and DWV-B as described previously (Kevill et al. 2017). The RT-qPCR program is as follows: 45 °C for 10 min and 95 °C for 10 min, followed by 35 cycles of 95 °C for 15 s, 58.5 °C (DWV-A and DWV-B) for 15 s, and 72 °C for 15 s. Each plate included an RNA standard curve, as described by Kevill et al. (2017), as well as a no-template control. Genomic equivalents per bee were calculated as described by Kevill et al. (2017).

### Pathogen Presence

To confirm diagnosis of EFB and to determine if nonsymptomatic colonies were exposed to *M. plutonius,* adult nurse bees were collected as they have been shown to have a more consistent level of *M. plutonius* in EFB-affected colonies when compared with larvae (Belloy et al. 2007). Adult nurse bees collected from the second timepoint (T2 –5th–6th June) were screened by culture for the presence of *M. plutonius* using a standard screen for adult bees (Roetschi et al. 2008), with slight modifications. A minimum of 15 adult nurse bees were added to a filtered grinding bag along with 0.5 ml phosphate-buffered saline (PBS) per adult bee and thoroughly macerated. Filtrate was transferred to 15 ml tubes and centrifuged at 1,150 g for 15 min. Supernatant (1 ml) was carefully pipetted off the resulting pellet and transferred to a sterile microcentrifuge tube. Multiple freeze thaw cycles of adult bee samples due to a freezer failure resulted in degradation, fungal overgrowth and subsequently PCR inhibition, so samples were cultured prior to undergoing PCR screening. Under anaerobic conditions, 1 µl of supernatant was streaked onto M110 agar (Forsgren et al. 2013) and incubated anaerobically at 37 °C for 5 days. Isolates previously preserved in glycerol stock, typical *M. plutonius* ATCC 35311 and a regional atypical *M. plutonius* isolate were cultured alongside samples as positive controls. Multiple colonies consistent with *M. plutonius* were collected using a sterile swab and transferred to 1 ml of sterile nuclease free water. After centrifuging at 10 k rpm for 3 min, supernatant was discarded, and the remaining pellet underwent DNA extraction using Instagene Matrix (BioRad) following the manufacturer’s instructions. Extracted DNA (2 µl) was used as template for a 25 µl duplex PCR screen as previously validated and described by Arai et al. (2014). Resulting amplicons were run on 1.2% gel at 90 V for 40 min, along with negative and positive controls and a 100 bp ladder (NEB). Bands at 187 bp were considered positive for typical *M. plutonius*, while bands at 424 bp were considered positive for atypical *M. plutonius*. The cultured regional atypical isolate and ATCC strain 35311 were used as positive controls for aytpical and typical strains, respectively for all PCR reactions. To further confirm the identity of bacterial species isolated, isolates obtained from screening were inoculated into 5 ml KSBHI broth anaerobically and incubated for 5 days at 37 °C, agitating daily. Broth culture was then centrifuged at 1,150 g for 10 min and supernatant was discarded. The pellet was resuspended in 1 ml of PBS and transferred to a 1.5 ml microcentrifuge tube. Samples were centrifuged at 10 k rpm for 3 min and supernatant was discarded. Resulting pellets were resuspended in 180 μl enzymatic lysis buffer (20 mM Tris-HCl, 2mM EDTA, 1.2% Triton X-100) containing 20 mg/mL lysozyme and incubated for 30 min at 56 °C. DNEasy Blood and Tissue kit (Qiagen, Valencia, CA) was used for extraction following the manufacturers protocols for gram-positive bacteria and eluted into 50 μl EB buffer. 2 μl of extracted DNA was used as template in a 12 μl PCR reaction using validated primers as described by Govan et al. 1998. PCR products were run on a 1.2% agarose gel at 90 V for 45 min along with a 100 kb ladder. A band at 810 bp was considered positive for *M. plutonius* (Govan et al. 1998).

### Statistical Analysis

All statistical analyses were carried out using R version 4.3.0 (R core team 2023). Differences in disease rates between yards were compared using a two-tailed Fischer exact test. To determine the impact of clinical EFB disease on colony growth the percent change in cluster between timepoint 1 and timepoint 3 was determined. For this analysis we removed colonies that had lost their queen at the third timepoint in order to remove that confounding variable. We additionally only included colonies that were diseased at the first 2 timepoints to ensure colonies had symptoms for a similar period and were not recently infected at timepoint 3. Relative risk of EFB on the ability of colonies to grow over the season was performed by categorizing each colony based on the change in cluster size between timepoint 1 and 3 (growth over the season) as “growth” or “no growth”. Colonies showing clinical EFB at timepoints 1 and 2 were placed in the exposure group, while colonies showing no growth or decline were placed in the negative outcome group. Normality of data was determined using Shapiro Wilks test. Comparisons between groups were done using one–way ANOVA or Welch two sample *t*-tests. Nonparametric alternative tests Kruskal–Wallis rank sum test and Wilcoxon rank sum test were used when the assumption of normality could not be satisfied. Alpha values of *P *≤ 0.05 were considered statistically significant. Visualizations were created using ggplot2 version 3.4.1 and edited in Adobe Illustrator version 24.1.2.

## Results

### Disease Prevalence Was High in Both Yards and Associated With the Presence of Atypical *M. plutonius*

**Fig. 1.**
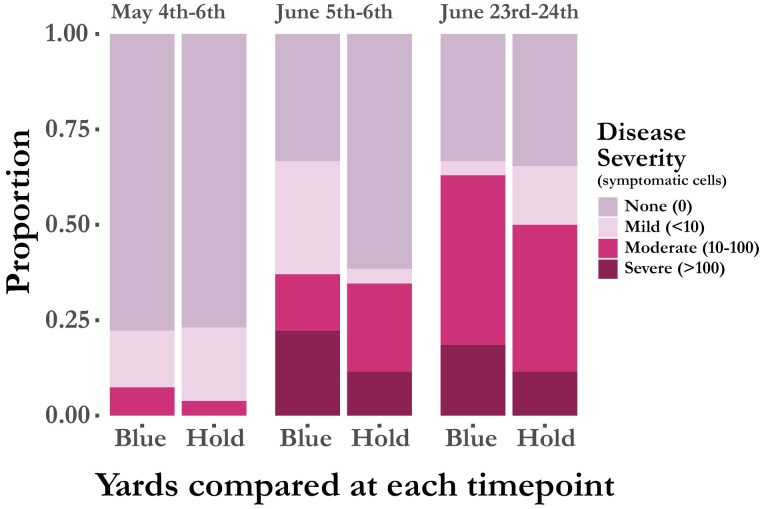
Percentage of colonies in each yard (Blueberry and Holding) that showed varying signs of European foulbrood at each timepoint. (None = No symptomatic cells; Mild = Less than 10 symptomatic cells; Moderate = between 10 and 100 symptomatic cells; Severe = Greater than 100 symptomatic cells).

Overall, we found significant levels of clinical EFB in both yards which increased in prevalence over the season. Over the course of the study, 62% (*n* = 33) of the colonies developed moderate to severe disease at some point. Disease progressed similarly in both yards (Fig. 1). At the initial inspection, T1 (4th–6th May), 2 of the 27 colonies in the blueberry yard and 1 colony in the holding yard were already showing moderate signs of disease. By the second timepoint, T2 (5th–6th June), 37% (*n* = 10) of the colonies in blueberry and 35% (*n* = 9) of the colonies in the holding yard had developed moderate to severe disease. At the final timepoint, T3 (23rd–24th June), more colonies had moderate to severe disease in the blueberry yard 63% (*n* = 17) than in the holding yard 50% (*n* = 13) but this difference was not statistically significant (*P* = 0.42; two-tailed Fisher exact test) with the current samples size.

Viable *M. plutonius* was recovered from 44 colonies. Screening revealed that all EFB-affected colonies (*n* = 33) had viable atypical *M. plutonius* present in adult nurse bees at the second timepoint, while an additional twelve colonies that never developed EFB also had viable atypical *M. plutonius* recovered. The remaining 9 colonies from which no *M. plutonius* was viable in adult nurse bees at timepoint 2, never developed EFB at any timepoint.

### Colonies That Had EFB Early in the Season Were More Likely to Show No Growth or Decrease in Size When Compared With Healthy Colonies

**Fig. 2.**
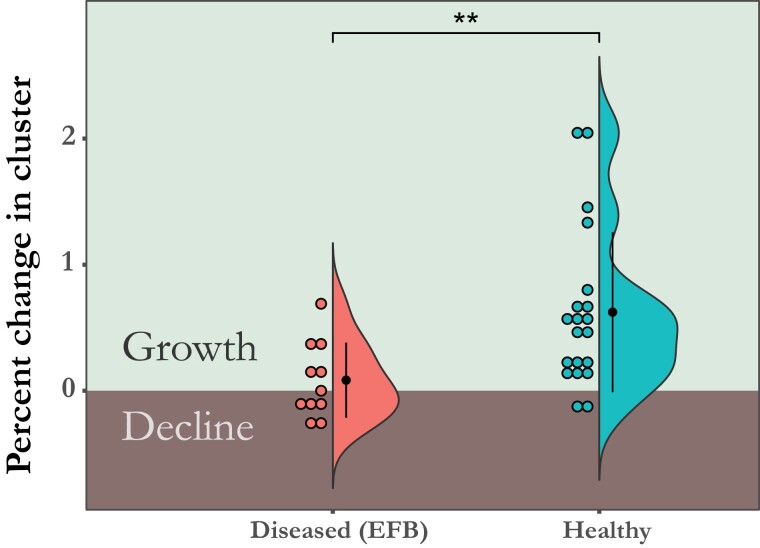
Comparison of cluster growth between T1 (4th–5th May) and T3 (25th–26th June) based on early symptoms of severe EFB. Cluster growth is expressed as a percentage of the starting size. Each dot represents a colony binned for each percent change in cluster size. The “Diseased” group are colonies that had severe EFB at timepoint 1 or 2. The “Healthy” colonies never developed any symptoms of EFB.

While our sample size limited our statistical power, we were able to show that early season EFB puts colonies at greater risk for stagnation or a decline in growth. Of the 48 colonies, 17 colonies had EFB by the second timepoint and 32 were healthy. Of the 16 colonies with EFB, 50% (*n* = 8) had no growth or negative growth over the course of the season. Of the 32 that had no disease, 15.6% (*n* = 5) had no growth or negative growth. Colonies with EFB were 3.2 times more likely to show no growth or decrease in size over the season when compared with healthy colonies (*P* = 0.01, 95% CI = 1.25–8.21, RR). Colonies with EFB had a (31%) reduction in mean growth when compared with healthy colonies, 18 and 49% respectively, but the difference between the 2 groups is not statistically significant (Wilcoxon rank sum; W = 188; *P* = 0.08) (Supplementary Fig. 1). Severely diseased colonies with more than 100 cells showing signs of EFB (*n* = 11) had a 53.9% reduction in colony growth when compared with colonies that never showed any clinical signs of EFB (*n* = 20) with 8.4 and 62.3% mean cluster change respectively (Wilcoxon rank sum; W = 45.5; *P* < 0.01) (Fig. 2). These colonies were 5.45 times more likely to decline in size or stagnate when compared to colonies that never showed any symptoms (*P* = 0.02, 95% CI = 1.32–22.60, RR).

### Hygienic Behavior Has No Impact on the Development of Clinical EFB in Infected Colonies

**Fig. 3.**
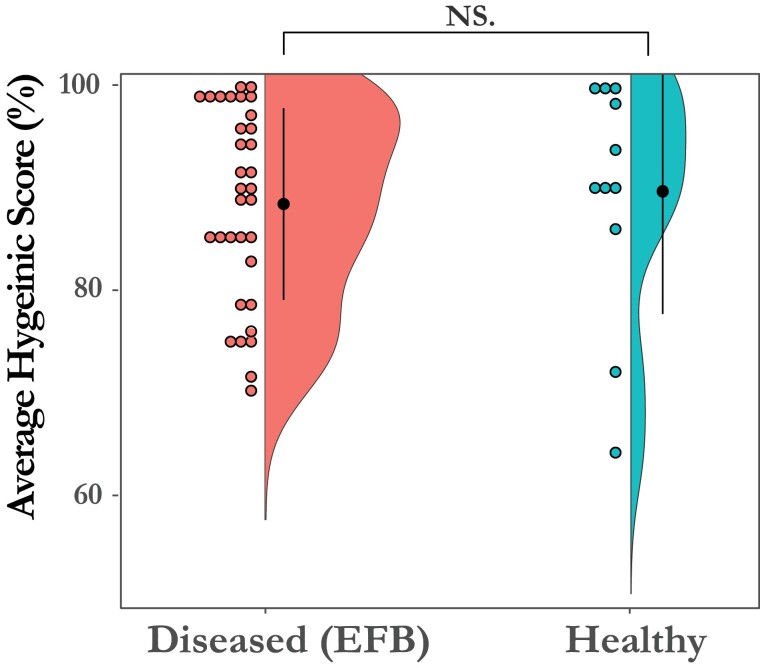
Colonies infected with *M. plutonius* that remained healthy did not have a significantly different hygienic score than those that developed clinical EFB disease over the course of the study. Each dot represents a colony; the y-axis represents the average partial hygienic score.

No differences were found in mean full or partial hygienic scores between EFB diseased colonies and *M. plutonius* infected colonies that never developed clinical disease. Average partial hygienic behavior for the 53 colonies varied between 51% and 100% with a mean of 89% (SD = 0.11), with 66% of the colonies (*n* = 34) having average hygienic scores below 95%, a common cutoff for considering a colony highly hygienic (Spivak and Danka 2021). Of the 44 colonies, 33 had clinical EFB, while 11 remained healthy for the entire study. Hygienic score for the diseased colonies varied between 70 and 100%, with a mean of 88% (SD = 9%—Shapiro-Wilk; W = 0.92; *P* = 0.01). Partial hygienic score for the infected, but healthy colonies ranged from 64% to 100% with a mean of 89% (SD = 11%—Shapiro-Wilk; W = 0.82; *P *= 0.01). The difference between the 2 means was not statistically significant (*P* = 0.80, Welch two sample *t*-test—two-tailed) (Fig. 3). We additionally found no difference when comparing the more conservative partial or full hygienic score on health outcome (Supplementary Fig. 2) and no significant difference between disease severity for full (Supplementary Fig. 3) or partial hygienic scores (Supplementary Fig. 4).

### Deformed Wing Virus (DWV) A and B Load Had No Effect on the Development of EFB Disease in *M. plutonius*-Infected Colonies

**Fig. 4.**
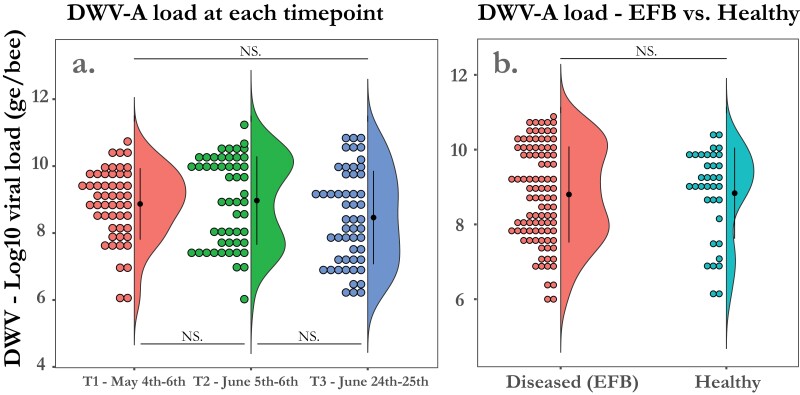
a) DWV-A loads did not change between timepoints. When comparing the mean DWV-A viral load as ge (Log10 genome copies per bee) in all 53 colonies in this study at each timepoint we found no significant difference. b) DWV-A loads did not differ between EFB diseased colonies and healthy colonies. When pooling both yards and comparing *M. plutonius* infected colonies that developed severe disease to those that remained healthy for the entire season, we found no difference in the average DWV-A viral load (Log10 DWV-A genome copies per bee). Each dot represents a colony binned by viral load to demonstrate the distribution in each group.

**Fig. 5.**
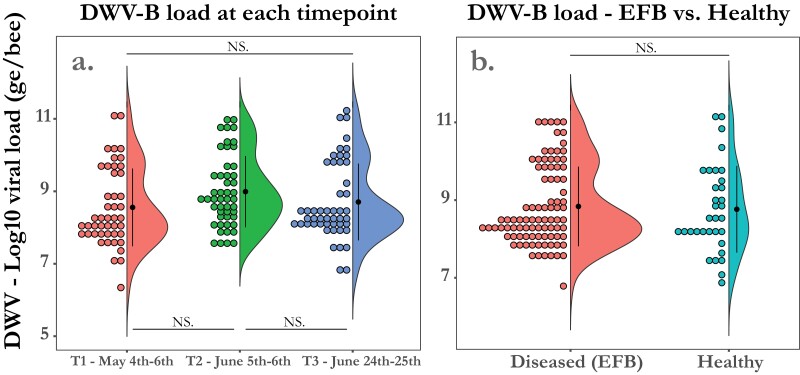
a) DWV-B loads increased at timepoint T2 (5th–6th June). When comparing the mean viral load as ge (Log10 genome copies per bee) in all 53 colonies in this study at each timepoint we found no significant difference between the first and last, or the second timepoint and the last, but did find an increase between the first and second timepoint. b) DWV-B loads did not differ between EFB diseased colonies and healthy colonies. When pooling both yards and comparing *M. plutonius* infected colonies that developed severe disease to those that remained healthy for the entire season, we found no difference in the average DWV-B viral load (Log10 DWV-B genome copies per bee). Each dot represents a colony binned by viral load to demonstrate the distribution in each group.

Among colonies where *M. plutonius* was found (*n* = 44), there was no difference in mean viral load for DWV-A or DWV-B between diseased colonies and healthy colonies. All 53 colonies tested had detectable levels of DWV-A at every timepoint ranging from 5.97 log_10_ to 11.23 log_10_ with a mean viral load of 8.77 log_10_ genome copies/bee (SD = 1.28). Mean viral load in genome copies per bee (ge) had no significant change between timepoints T1—4th–6th May (mean = 8.87 log_10_), T2—5th–6th June (mean = 8.97 log_10_), or T3—23rd–24th June (mean = 8.46 log_10_) (one-way ANOVA; *F* = 2.49; df = 2; *P* = 0.09) (Fig. 4a). Additionally, there was no significant difference between the mean viral load (genome copies per bee) in *M. plutonius* infected colonies that developed clinical symptoms of EFB (mean = 8.81 log_10_) when compared with *M. plutonius* infected colonies that remained healthy over the course of the study (mean = 8.95 log_10_) (Wilcoxon rank sum test; W = 1,643 *P* = 0.92) (Fig. 4b). All but 5 colonies, 3 from timepoint T1—4th–6th May and 2 additional colonies from timepoint T2—5th–6th June, had detectable levels of DWV-B at every timepoint. The loads of DWV-B in the colonies with detectable levels ranged from 6.34 log_10_ to 11.23 log_10_ with a mean of 8.76 log_10_ genome copies/bee (SD = 1.05). There was no significant change in mean DWV-B genome copies per bee between timepoint T1—4th–6th May (mean = 8.57 log_10_), T2—5th–6th June (mean = 8.99 log_10_), and T3 (mean = 8.71 log_10_) (one-way ANOVA; *F *= 2.40; *P* = 0.09) (Fig. 5a). When comparing *M. plutonius* infected colonies that developed disease with those that never developed disease we also found no significant difference in the mean DWV-B genome copies per bee, 8.84 log_10_ and 8.76 log_10_, respectively (Wilcoxon rank sum test; W = 1531; *P* = 0.83) (Fig. 5b). Twelve *M. plutonius* infected colonies had very high viral loads for DWV-A and B exceeding 9 log_10_ for both viruses. Seven of them developed EFB and 5 never developed any EFB signs.

## Discussion

This study revealed a high rate of EFB in both yards with atypical strains of *M. plutonius* identified. Additionally, we showed that these strains of *M. plutonius* remained viable and were easily recovered from adult nurse bees in all diseased colonies as well as many colonies that never developed disease. Infected colonies that did develop disease were at a significantly greater risk of stagnation or decline than healthy colonies, which highlights the importance of this disease. We additionally found no relationship between the development of EFB in *M. plutonius* infected colonies and their concurrent viral load of DWV-A or DWV-B. Finally, while hygienic behavior has shown to be effective in managing AFB, there does not seem to be any benefit when considering the risk of EFB.

### Impact of Viral Coinfection

Interestingly, mean viral load in all colonies had little variation for either virus (DWV-A or DWV-B) over the 3 timepoints (T1—4th–5th May, T2—5th–6th June, T3—23rd–24th June) in this study and did not appear to impact the development of clinical EFB. While we expected colonies with high early viral loads to be more susceptible to EFB, we found no relationship with colonies that had high viral loads for DWV-A or DWV-B with the development of clinical EFB over the course of the season. One colony where viable *M. plutonius* was detected along with high viral loads for both DWV-A (9.34 log_10_ genome copies per bee) and DWV-B (11.1 log_10_ genome copies per bee) at timepoint 1 never developed EFB, while another colony with comparatively low viral loads of, 5.97 log_10_ genome copies per bee and 8.1 log_10_ genome copies per bee respectively, developed severe EFB. Additionally, it does not seem that the development of EFB leaves colonies more susceptible to DWV as colonies that became sick with EFB at the final timepoint had no significant difference in viral load when compared with those that remained healthy the entire season. However, because DWV-A was found in every colony in the study and DWV-B was found in all but 5, it is difficult to draw a conclusion about the role of subclinical DWV infection on EFB development as previous research has reported clinical pathology at viral levels well below our mean (Koziy et al. 2019). It should also be noted that DWV levels of adult nurse bees may be different than the levels in larvae and may not reveal an increased amount of viral replication due to EFB in affected larvae. Additionally, it should be noted that qPCR used in this study is unable to resolve the complex of viral variants and subtypes present in the samples (de Miranda et al. 2022).

### Hygienic Behavior

Hygienic behavior has been shown to be a beneficial trait for American foulbrood (Spivak and Gilliam 1998, Spivak and Reuter 2001), chalkbrood (Palacio et al. 2010), and the *Varroa* mite (Spivak and Danka 2021), however our results suggest that it has no impact on the development of EFB. The colony with the lowest average partial hygienic score in the study (64.2%) remained healthy the entire season, despite having viable *M. plutonius* present, while 2 of the 3 colonies that were severely diseased the entire season had very high hygienic scores (98.6%, 98.9%). Overall, average partial hygienic behavior was widely distributed around the typical cutoff of 90–95% (Spivak and Downey 1998, Boutin et al. 2015). However, testing colonies with more substantial differences in hygienic behavior and with a larger sample size may yield different results.

While it was surprising to find no impact of partial hygienic behavior on the development of EFB, it is consistent with previous research (Milne 1985) and may be related to pathogen virulence and lifecycle. One of the key features of hygienic behavior measured by our assay is the ability of adult bees to detect dead capped pupae, uncap these cells and quickly remove the pupae. This is useful for preventing American foulbrood (AFB), as *Paenibacillus larvae* (White) (Bacillales: Paenibacillaceae) during the early, vegetative stages of disease is not infectious and can be removed before drying down into a scale containing infectious spores (Djukic et al. 2014). On the other hand, *M. plutonius* is infectious at all stages so it is uncertain whether behavior that increases the removal of diseased larvae would aid by reducing the bacterial load in the colony, or whether it would make the disease worse by spreading infectious bacteria around the hive. Additionally, AFB is known to affect larvae at a later stage of development than European foulbrood and detecting and removing dead pupae under cappings at this later stage may not confer the same behavior to diseased brood at earlier stages. This is corroborated by similar research on the impact of hygienic behavior on sacbrood virus (Choi et al. 2022). It should be noted that only atypical *M. plutonius* was identified in this study which is known to increase early larval mortality significantly and these findings may not be relevant for slower growing typical strains (Grossar et al. 2020, Nakamura et al. 2020). Further research making use of multiple strains of *M. plutonius* and the development of an open brood removal assay may provide additional insights into genetic behaviors related to EFB resistance.

### Impact of EFB on Colony Growth

While sample size is small, we were able to show a significant impact in the growth of the colony due to atypical EFB. Mean percent growth of severely EFB diseased colonies over the season was reduced by 54% and these colonies were 5.5 times more likely (*P* = 0.02) to show no growth or a decline in population when compared to colonies that remained healthy. However, this study only followed colonies through the end of June and long-term impact on colony health and honey production is still unclear. Very few studies have examined the long term impact that EFB has on colony health and growth over the season. Repeating this study with a larger sample size and multiple yards would help increase the power of the analysis and provide a more detailed picture of the economic impact of this disease at differing levels of severity.

Here we report the progression and impact of EFB caused by atypical *M. plutonius* among honey bee colonies at 2 sites from a single commercial operation in Michigan over the course of the season from 4th May to—25th June. We failed to find any relationship between the viral load of DWV-A or DWV-B and the progression of EFB in infected colonies. However, we were only able to include 2 sites from a single beekeeper, so the impact of other confounding factors such as weather, location, nutrition, other viral variants, and coinfections as well as host genetics remains unclear. Including more sites, larger sample sizes and metagenomic analysis in future studies may help confirm our findings. Unfortunately, we were also unable to find any benefit of hygienic behavior on the development of EFB, however the presence of only atypical *M. plutonius* at our sites as well as the limitations of our hygienics assay to determine hygienic behavior toward open brood merit further study. While few studies have focused on EFB in the United States, the high rates of disease reported here and elsewhere (Grant et al. 2021) and the reported risk to colony growth make additional research essential for the long-term sustainability of the bee keeping industry.

## Supplementary Material

iead094_suppl_Supplementary_Figures_S1-S4

## References

[CIT0002] Arai R , Tominaga K, Wu M, Okura M, Ito K, Okamura N, Onishi H, Osaki M, Sugimura Y, Yoshiyama M, et al. Diversity of *Melissococcus plutonius* from honeybee larvae in Japan and experimental reproduction of European foulbrood with cultured atypical isolates. PLoS One. 2012:7(3):e33708–e33710. 10.1371/journal.pone.0033708 22442715 PMC3307753

[CIT0005] Belloy L , Imdorf A, Fries I, Forsgren E, Berthoud H, Kuhn R, Charrière JD. Spatial distribution of *Melissococcus plutonius* in adult honey bees collected from apiaries and colonies with and without symptoms of European foulbrood. Apidologie. 2007:38(2):136–140. 10.1051/apido:2006069

[CIT0006] Benaets K , Van Geystelen A, Cardoen D, De Smet L, De Graaf DC, Schoofs L, Larmuseau MHD, Brettell LE, Martin SJ, Wenseleers T. Covert deformed wing virus infections have long-term deleterious effects on honeybee foraging and survival. Proc R Soc B Biol Sci. 2017:284(1848):20162149. 10.1098/rspb.2016.2149 PMC5310602 28148747

[CIT0007] Boutin S , Alburaki M, Mercier PL, Giovenazzo P, Derome N. Differential gene expression between hygienic and non-hygienic honeybee (*Apis mellifera* L) hives. BMC Genomics. 2015:16(1):1–13. 26149072 10.1186/s12864-015-1714-y PMC4491870

[CIT0008] Bruckner S , Wilson M, Aurell D, Rennich K, vanEngelsdorp D, Steinhauer N, Williams GR. A national survey of managed honey bee colony losses in the USA: results from the Bee Informed Partnership for 2017–18, 2018–19, and 2019–20. J Apic Res. 2022:62(3):429–443. 10.1080/00218839.2022.2158586

[CIT0009] Budge GE , Barrett B, Jones B, Pietravalle S, Marris G, Chantawannakul P, Thwaites R, Hall J, Cuthbertson AGS, Brown MA. The occurrence of *Melissococcus plutonius* in healthy colonies of *Apis mellifera* and the efficacy of European foulbrood control measures. J Invertebr Pathol. 2010:105:164–170. 20600088 10.1016/j.jip.2010.06.004

[CIT0010] Choi YS , Geun PH, Frunze O. Differential hygienic behavior of *Apis cerana* F. and *Apis mellifera* L to Sacbrood virus infection. J Asia-Pac Entomol. 2022:25(4):101995. 10.1016/j.aspen.2022.101995

[CIT0011] de León-Door AP , Romo-Chacón A, Rios-Velasco C, Zamudio-Flores PB, Ornelas-Paz J. de J., Acosta-Muñiz CH. Prevalence, typing and phylogenetic analysis of *Melissococcus plutonius* strains from bee colonies of the State of Chihuahua, Mexico. J Invertebr Pathol. 2018:159:71–77. 10.1016/j.jip.2018.10.006 30312627

[CIT0012] de Miranda JR , Brettell LE, Chejanovsky N, Childers AK, Dalmon A, Deboutte W, de Graaf DC, Doublet V, Gebremedhn H, Genersch E, et al. Cold case: the disappearance of Egypt bee virus, a fourth distinct master strain of deformed wing virus linked to honeybee mortality in 1970’s Egypt. Virol J. 2022:19(1):1–11. 35033134 10.1186/s12985-022-01740-2 PMC8760790

[CIT0013] de Miranda JR , Genersch E. Deformed wing virus. J Invertebr Pathol. 2010:103(Suppl 1):S48–S61. 10.1016/j.jip.2009.06.012 19909976

[CIT0014] Djukic M , Brzuszkiewicz E, Fünfhaus A, Voss J, Gollnow K, Poppinga L, Liesegang H, Garcia-Gonzalez E, Genersch E, Daniel R. How to kill the honey bee larva: genomic potential and virulence mechanisms of *Paenibacillus larvae*. PLoS One. 2014:9(3):e90914. 10.1371/journal.pone.0090914 24599066 PMC3944939

[CIT0015] Erban T , Ledvinka O, Kamler M, Hortova B, Nesvorna M, Tyl J, Titera D, Markovic M, Hubert J. European foulbrood in Czechia after 40 years: application of next-generation sequencing to analyze *Melissococcus plutonius* transmission and influence on the bacteriome of *Apis mellifera*. PeerJ Prepr. 2016:4:e2618–e26v1.

[CIT0016] Floyd AS , Mott BM, Maes P, Copeland DC, McFrederick QS, Anderson KE. Microbial ecology of European foul brood disease in the honey bee (*Apis mellifera*): towards a microbiome understanding of disease susceptibility. Insects. 2020:11(9):555–516. 10.3390/insects11090555 32825355 PMC7565670

[CIT0017] Forsgren E , Budge GE, Charrière JD, Hornitzky MAZ. Standard methods for European foulbrood research. J Apic Res. 2013:52(1):1–14. 10.3896/ibra.1.52.1.12

[CIT0018] Gisder S , Möckel N, Eisenhardt D, Genersch E. In vivo evolution of viral virulence: switching of deformed wing virus between hosts results in virulence changes and sequence shifts. Environ Microbiol. 2018:20(12):4612–4628. 10.1111/1462-2920.14481 30452113

[CIT0019] Govan VA , Brözel V, Allsopp MH, Davison S. A PCR detection method for rapid identification of *Melissococcus pluton* in honeybee larvae. Appl Environ Microbiol. 1998:64(5):1983–1985. 10.1128/AEM.64.5.1983-1985.1998 9572987 PMC106266

[CIT0020] Grant KJ , DeVetter L, Melathopoulos A. Honey bee (*Apis mellifera*) colony strength and its effects on pollination and yield in highbush blueberries (*Vaccinium corymbosum*). PeerJ. 2021:9:e11634–e11618. 10.7717/peerj.11634 34395063 PMC8323595

[CIT0021] Grossar D , Kilchenmann V, Forsgren E, Charrière JD, Gauthier L, Chapuisat M, Dietemann V. Putative determinants of virulence in *Melissococcus plutonius*, the bacterial agent causing European foulbrood in honey bees. Virulence. 2020:11(1):554–567. 10.1080/21505594.2020.1768338 32456539 PMC7567439

[CIT0022] Highfield AC , El Nagar A, Mackinder LCM, Noël LMLJ, Hall MJ, Martin SJ, Schroeder DC. Deformed wing virus implicated in overwintering honeybee colony losses. Appl Environ Microbiol. 2009:75(22):7212–7220. 10.1128/aem.02227-09 19783750 PMC2786540

[CIT0023] Iqbal J , Mueller U. Virus infection causes specific learning deficits in honeybee foragers. Proc Biol Sci. 2007:274(1617):1517–1521. 10.1098/rspb.2007.0022 17439851 PMC2176156

[CIT0024] Kathe E , Seidelmann K, Lewkowski O, Le Conte Y, Erler S. Changes in chemical cues of *Melissococcus plutonius* infected honey bee larvae. Chemoecology. 2021:31(3):189–200. 10.1007/s00049-021-00339-3

[CIT0025] Keel CC. The Buzz About Pollinators. USDA Blog; 2022. [accessed 2023 Dec 7]. https://www.usda.gov/media/blog/2022/06/22/buzz-about-pollinators

[CIT0026] Kevill JL , de Souza FS, Sharples C, Oliver R, Schroeder DC, Martin SJ. DWV-A lethal to honey bees (*Apis mellifera*): a colony level survey of DWV Variants (A, B, and C) in England, Wales, and 32 States across the US. Viruses. 2019:11(5):426. 10.3390/v11050426 31075870 PMC6563202

[CIT0027] Kevill JL , Highfield A, Mordecai GJ, Martin SJ, Schroeder DC. ABC assay: Method development and application to quantify the role of three DWV master variants in overwinter colony losses of European honey bees. Viruses. 2017:9:1–14. 10.3390/v9110314 PMC5707521 29077069

[CIT0028] Koziy RV , Wood SC, Kozii IV, van Rensburg CJ, Moshynskyy I, Dvylyuk I, Simko E. Deformed wing virus infection in honey bees (*Apis mellifera* L). Vet Pathol. 2019:56(4):636–641. 10.1177/0300985819834617 30857499

[CIT0029] Laate EA , Emunu JP, Duering A, Ovinge L, Lynae O. Potential economic impact of European and American foulbrood on Alberta’s beekeeping industry; 2020. https://open.alberta.ca/dataset/029a345b-8621-4986-ad78-7fc6ddcd8b17/resource/25f7b78d-a359-428c-9648-9175c3634720/download/af-potential-economic-impact-european-american-foulbrood-on-albertas-beekeeping-industry.pdf

[CIT0030] Lanzi G , de Miranda JR, Boniotti MB, Cameron CE, Lavazza A, Capucci L, Camazine SM, Rossi C. Molecular and biological characterization of deformed wing virus of honeybees (*Apis mellifera* L). J Virol. 2006:80(10):–5009. 10.1128/JVI.80.10.4998-5009.2006 PMC1472076 16641291

[CIT0031] Lapidge KL , Oldroyd BP, Spivak M. Seven suggestive quantitative trait loci influence hygienic behavior of honey bees. Naturwissenschaften. 2002:89(12):565–568. 10.1007/s00114-002-0371-6 12536279

[CIT0032] Masood F , Thebeau JM, Cloet A, Kozii IV, Zabrodski MW, Biganski S, Liang J, Marta Guarna M, Simko E, Ruzzini A, et al. Evaluating approved and alternative treatments against an oxytetracycline-resistant bacterium responsible for European foulbrood disease in honey bees. Sci Rep. 2022:12(1):5906. 10.1038/s41598-022-09796-4 35393467 PMC8991240

[CIT0033] McKee BA , David Goodman R, Alan Hornitzky M. The transmission of European foulbrood (*Melissococcus plutonius*) to artificially reared honey bee larvae (*Apis mellifera*). J Apic Res. 2004:43(3):93–100. 10.1080/00218839.2004.11101117

[CIT0034] Milbrath MOG , Fowler PD, Abban SK, Lopez D, Evans JD. Validation of diagnostic methods for European foulbrood on commercial honey bee colonies in the United States. J Insect Sci. 2021:21. 10.1093/jisesa/ieab075 PMC8559156 34723329

[CIT0035] Milne CPJ. Laboratory tests to honey bee hygienic behavior and resistance to European foulbrood. Am Bee J. 1985:125(8):578–580.

[CIT0036] Nakamura K , Okumura K, Harada M, Okamoto M, Okura M, Takamatsu D. Different impacts of pMP19 on the virulence of *Melissococcus plutonius* strains with different genetic backgrounds. Environ Microbiol. 2020:22(7):2756–2770. 10.1111/1462-2920.14999 32219986

[CIT0037] Palacio MA , Rodriguez E, Goncalves L, Bedascarrasbure E, Spivak M. Hygienic behaviors of honey bees in response to brood experimentally pin-killed or infected with *Ascosphaera apis*. Apidologie. 2010:41(6):602–612. 10.1051/apido/2010022

[CIT0038] Penn HJ , Simone-Finstrom MD, Chen Y, Healy KB. Honey bee genetic stock determines deformed wing virus symptom severity but not viral load or dissemination following pupal exposure. Front Genet. 2022:13:1–19. 10.3389/fgene.2022.909392 PMC9204523 35719388

[CIT0039] Phillips EF. The control of European foul brood. (No. 975). U.S. Dept. of Agriculture, Bureau of Entomology, Washington D. C., USA; 1918.

[CIT0040] Piou V , Schurr F, Dubois E, Vétillard A. Transmission of deformed wing virus between Varroa destructor foundresses, mite offspring and infested honey bees. Parasites Vectors. 2022:15(1): 1–15. 36151583 10.1186/s13071-022-05463-9 PMC9502634

[CIT0041] R Core Team. R: a language and environment for statistical computing. Vienna (Austria): R foundation for Statistical Computing; 2023.

[CIT0042] Richards ED , Tell LA, Davis JL, Baynes RE, Lin Z, Maunsell FP, Riviere JE, Jaberi-Douraki M, Martin KL, Davidson G. Honey bee medicine for veterinarians and guidance for avoiding violative chemical residues in honey. J Am Vet Med Assoc. 2021:259(8):860–873. 10.2460/javma.259.8.860 34609191

[CIT0043] Roetschi A , Berthoud H, Kuhn R, Imdorf A. Infection rate based on quantitative real-time PCR of *Melissococcus plutonius*, the causal agent of European foulbrood, in honeybee colonies before and after apiary sanitation. Apidologie. 2008:39(3):362–371. 10.1051/apido:200819

[CIT0044] Schroeder DC , Martin SJ. Deformed wing virus: the main suspect in unexplained honeybee deaths worldwide. Virulence. 2012:3(7):589–591. 10.4161/viru.22219 23154287 PMC3545936

[CIT0045] Spivak M , Danka RG. Perspectives on hygienic behavior in *Apis mellifera* and other social insects. Apidologie. 2021:52(1):1–16. 10.1007/s13592-020-00784-z

[CIT0046] Spivak M , Downey DL. Field assays for hygienic behavior in honey bees (Hymenoptera: Apidae). J Econ Entomol. 1998:91(1):64–70. 10.1093/jee/91.1.64

[CIT0047] Spivak M , Gilliam M. Hygienic behaviour of honey bees and its application for control of brood diseases and varroa: part II studies on hygienic behaviour since the Rothenbuhler era. Bee World. 1998:79(4):169–186. 10.1080/0005772x.1998.11099408

[CIT0048] Spivak M , Reuter GS. Performance of hygienic honey bee colonies in a commercial apiary. Apidologie. 1998:29(3):291–302. 10.1051/apido:19980308

[CIT0049] Spivak M , Reuter GS. Resistance to American foulbrood disease by honey bee colonies *Apis mellifera* bred for hygienic behavior. Apidologie. 2001:32(6):555–565. 10.1051/apido:2001103

[CIT0052] Traniello IM , Bukhari SA, Kevill J, Ahmed AC, Hamilton AR, Naeger NL, Schroeder DC, Robinson GE. Meta-analysis of honey bee neurogenomic response links deformed wing virus type A to precocious behavioral maturation. Sci Rep. 2020:10(1):1–12. 32080242 10.1038/s41598-020-59808-4 PMC7033282

[CIT0053] Wardell GI. European foulbrood: association with Michigan blueberry pollination, and control [Ph.D. dissertation]. East Lansing, Michigan, USA: Michigan State University; 1982.

[CIT0054] White GF. The cause of E uropean foul brood. No. 157. U.S. Dept. of Agriculture, Bureau of Entomology, Washington D. C., USA; 1912.

[CIT0055] White GF. European foulbrood. No. 810. U.S. Dept. of Agriculture, Bureau of Entomology, Washington D. C., USA; 1920.

[CIT0056] Wood SC , Chalifour JC, Kozii IV, de Mattos IM, Klein CD, Zabrodski MW, Moshynskyy I, Guarna MM, Veiga PW, Epp T, et al. In vitro effects of pesticides on European foulbrood in honeybee larvae. Insects. 2020:11:1–14. 10.3390/insects11040252 PMC7240397 32316434

