# Supplementary material for: No impact of hygienic behavior and viral coinfection on the development of European foulbrood in honey bee (Apis mellifera) colonies during blueberry pollination in Michigan
Source: J Insect Sci. 2023 Dec 6;23(6):21. doi: 10.1093/jisesa/iead094 (PMC10699875; doi:10.1093/jisesa/iead094)
Supplement: iead094_suppl_Supplementary_Figures_S1-S4 [file iead094_suppl_supplementary_figures_s1-s4.docx]

**Supplemental material**

**No impact of hygienic behavior and viral coinfection on the development of European foulbrood in honey bee (*Apis mellifera*) colonies during blueberry pollination in Michigan**

**
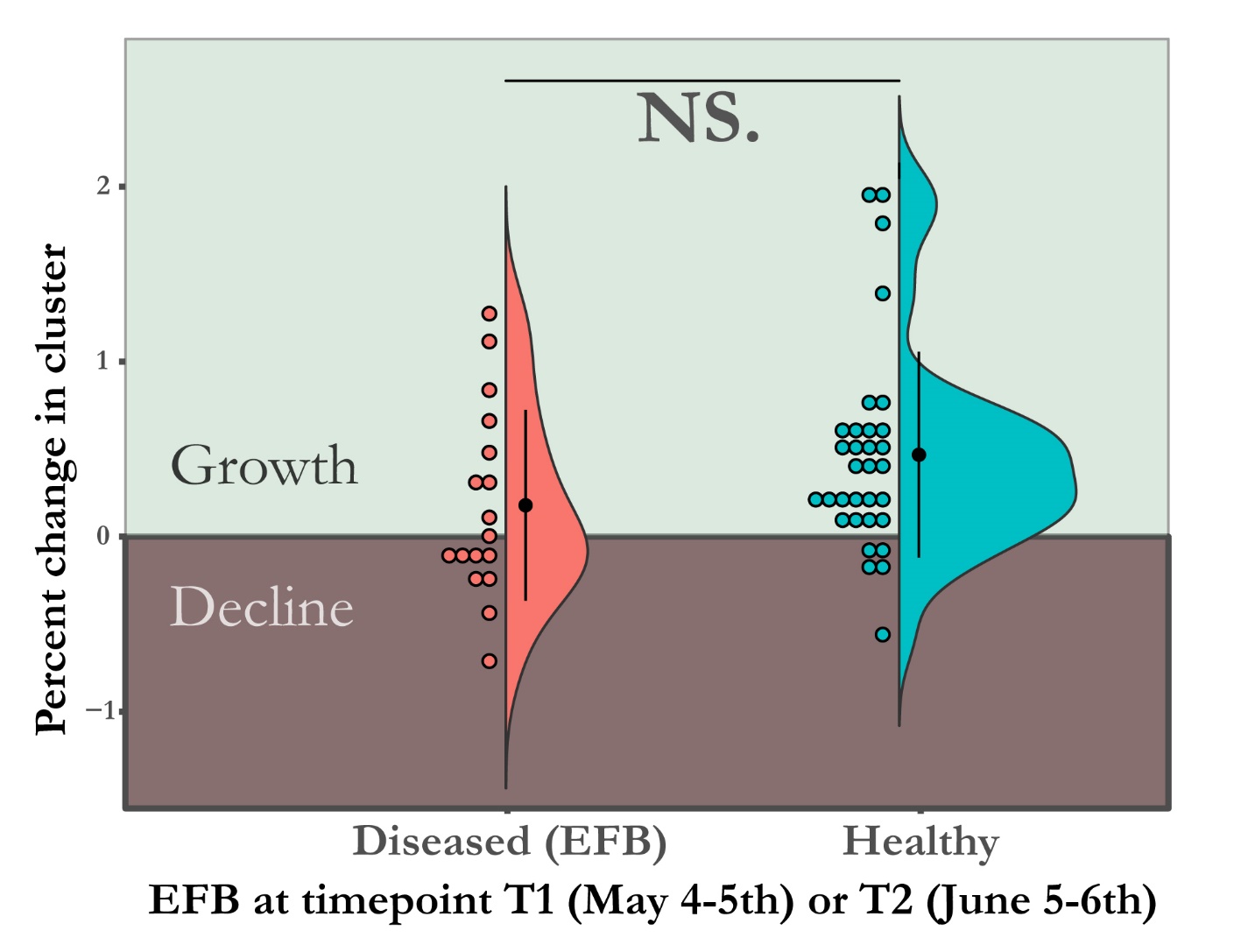
**

**Fig. S1** – Comparison of cluster growth between T1 (May 4-5^th^) and T3 (June 25^th^-26^th^) based on early symptoms of EFB (colonies showing signs at timepoint 1 or 2). Each dot represents a colony binned for each percent change in cluster size.


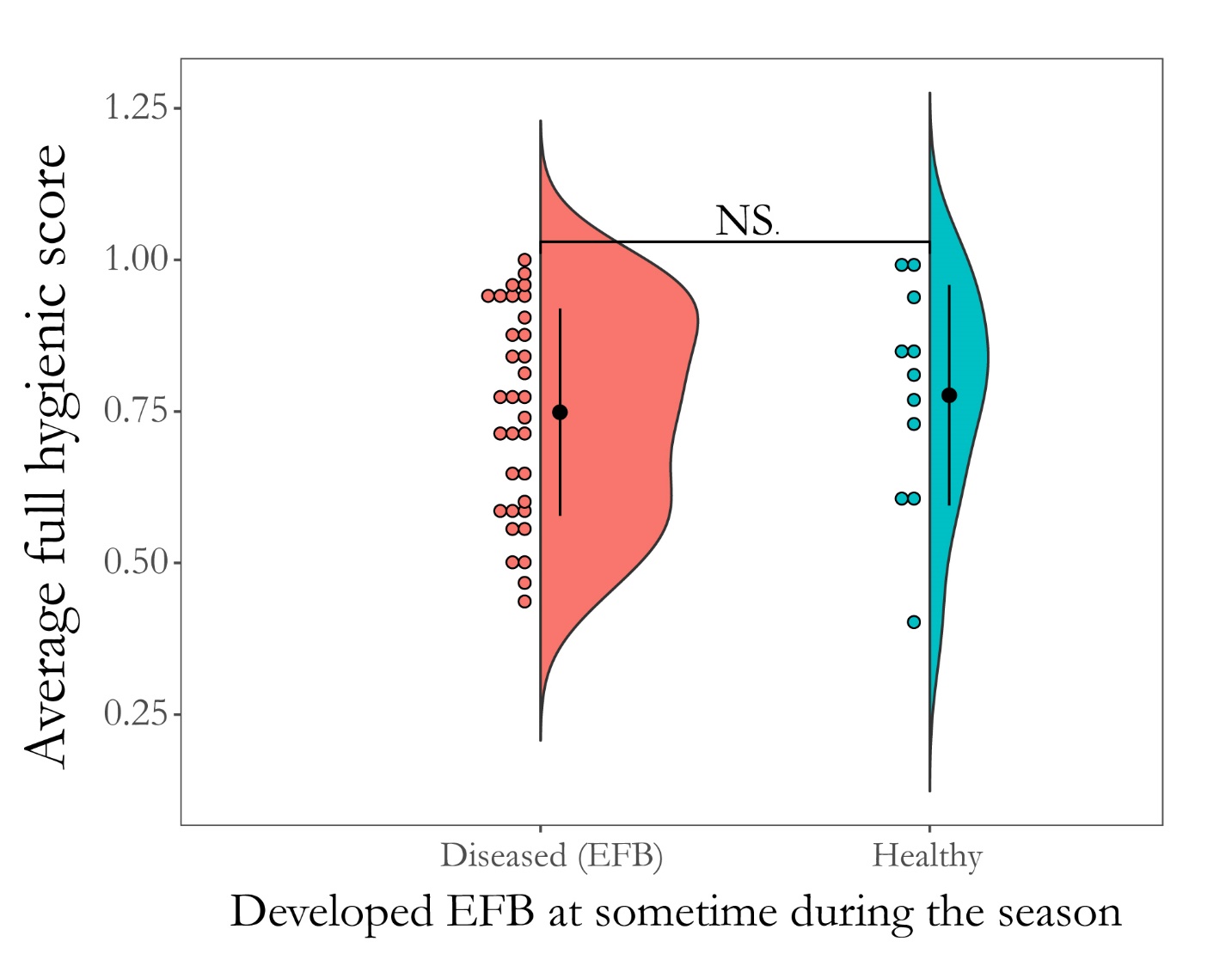


**Fig. S2** – Average full hygienic score for *M. plutonius* exposed colonies shows no difference between colonies that developed EFB (Diseased) and colonies that remained disease free the entire season (Healthy). Each dot represents a colony binned for average full hygienic score as measured by percentage of dead pupae completely removed after 24 hours.


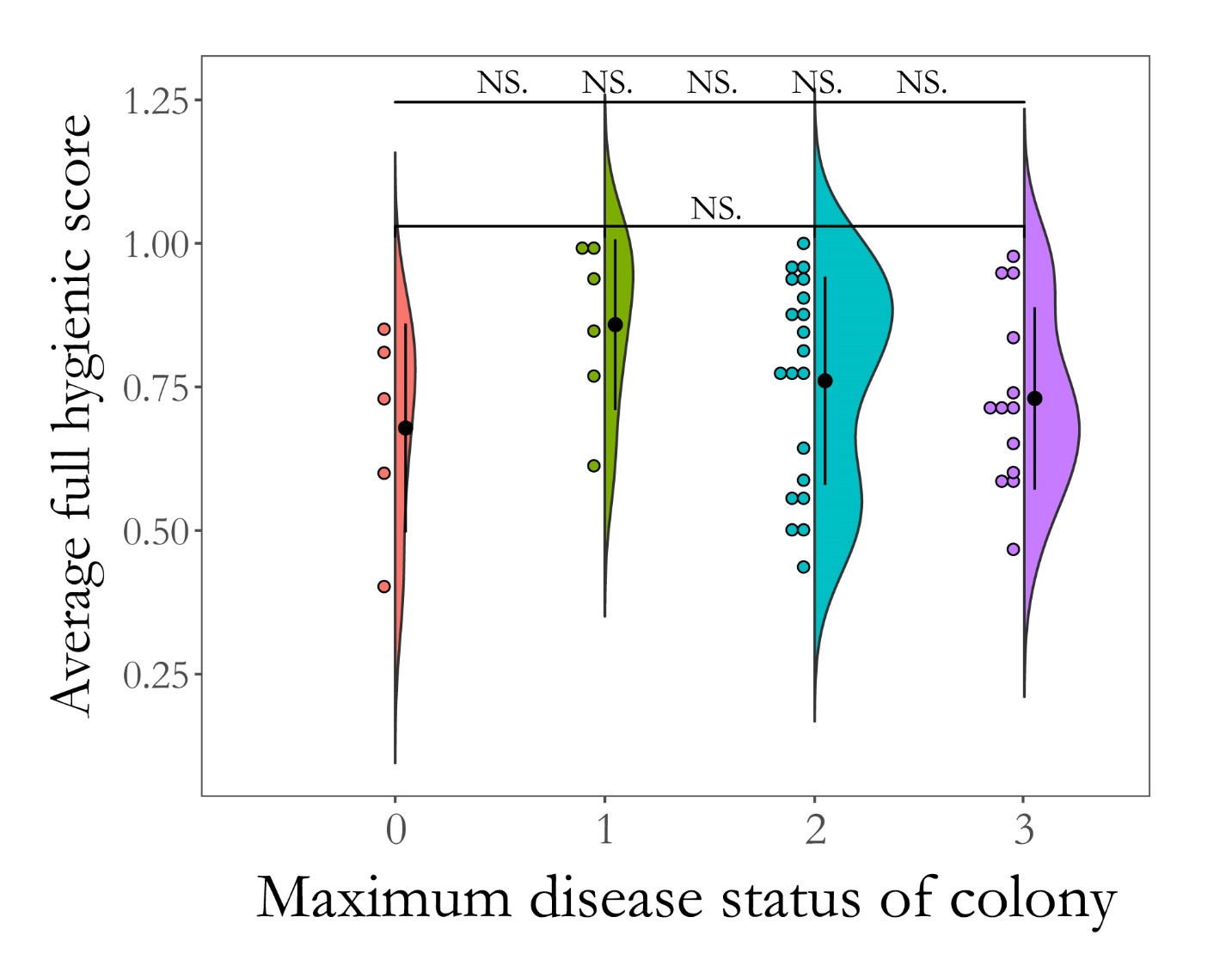


**Fig. S3** – Average full hygienic score for *M. plutonius* exposed colonies shows no difference between any level of EFB severity reached by each colony over the season. Severity is classified as follows: 0 = no symptomatic cells, 1 = < 10 symptomatic cells, 2 = 10-100 symptomatic cells, 3 = >100 symptomatic cells. Each dot represents a colony binned for average full hygienic score as measured by percentage of dead pupae completely removed after 24 hours.


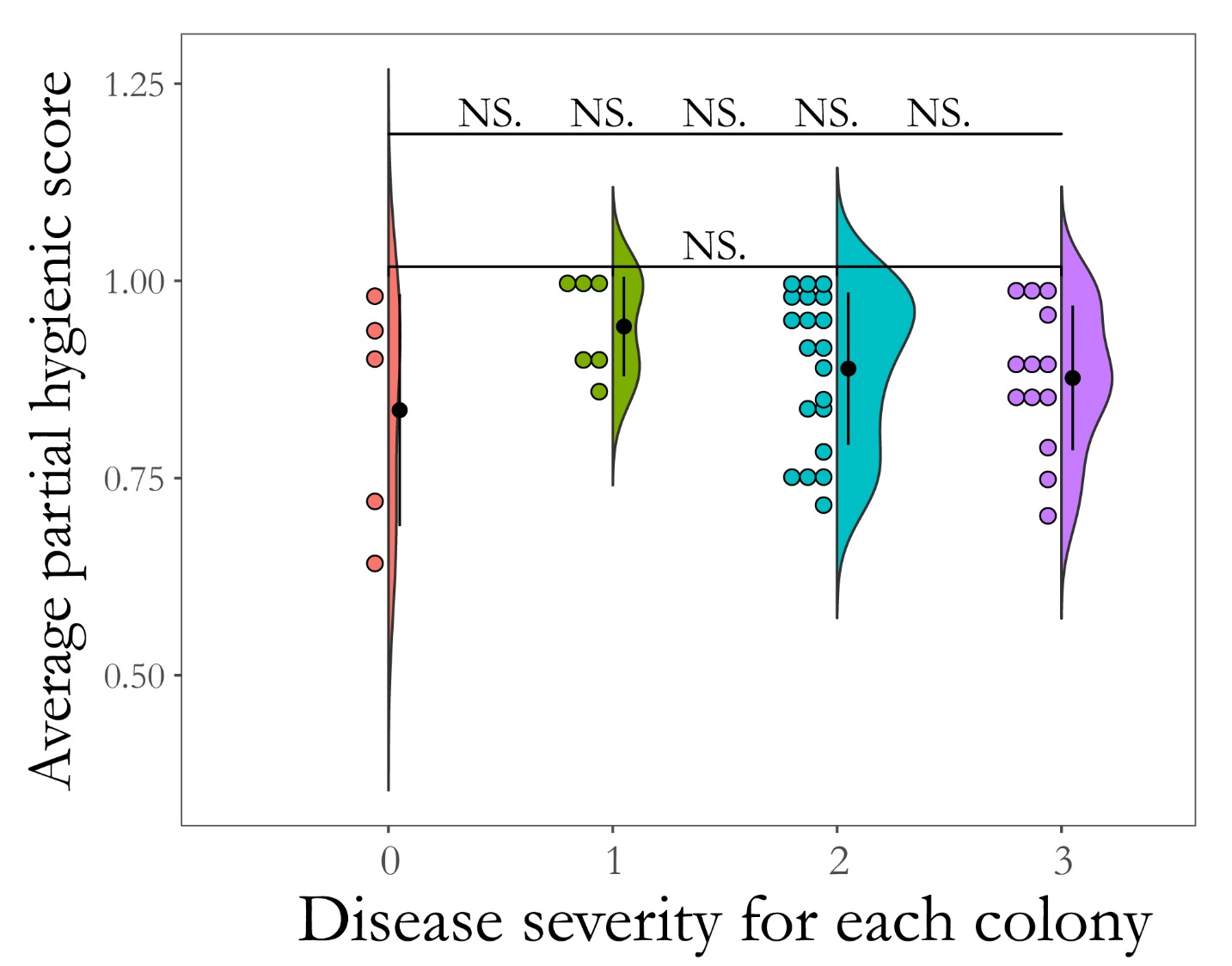


**Fig. S4** – Average partial hygienic score for *M. plutonius* exposed colonies shows no difference between any level of EFB severity reached by each colony over the season. Severity is classified as follows: 0 = no symptomatic cells, 1 = < 10 symptomatic cells, 2 = 10-100 symptomatic cells, 3 = >100 symptomatic cells. Each dot represents a colony binned for average partial hygienic score as measured by percentage of dead pupae uncapped or removed completely after 24 hours.

**Mite levels – Mean = 0.38 mites per 100 bees**

**Mite counts were performed by alcohol wash on 41 of the 53 colonies and reported here as mites per 100 bees**

**21 colonies = 0 mites**

**9 colonies = 0.3 mites**

**4 colonies = 0.7 mites**

**4 colonies = 1 mite**

**1 colony = 1.7 mites**

**1 colony = 2 mites**

**1 colony = 2.3 mites**
